# Exploiting differential Wnt target gene expression to generate a molecular biomarker for colorectal cancer stratification

**DOI:** 10.1136/gutjnl-2019-319126

**Published:** 2019-09-28

**Authors:** Sam O Kleeman, Viktor H Koelzer, Helen JS Jones, Ester Gil Vazquez, Hayley Davis, James E East, Roland Arnold, Martijn AJ Koppens, Andrew Blake, Enric Domingo, Chris Cunningham, Andrew D Beggs, Valerie Pestinger, Maurice B Loughrey, Lai-Mun Wang, Tamsin RM Lannagan, Susan L Woods, Daniel Worthley, S:CORT Consortium, Ian Tomlinson, Philip D Dunne, Timothy Maughan, Simon J Leedham

**Affiliations:** 1 Intestinal Stem Cell Biology Lab, Wellcome Trust Centre Human Genetics, University of Oxford, Oxford, UK; 2 Department of Pathology and Molecular Pathology, University Hospital Zürich, Zurich, Switzerland; 3 Oxford Colorectal Surgery Department, Nuffield Department of Surgery, Churchill Hospital, Oxford, Oxfordshire, UK; 4 Translational Gastroenterology Unit, John Radcliffe Hospital, Oxford, UK; 5 Cancer Genetics and Evolution Laboratory, Institute of Cancer and Genomic Sciences, University of Birmingham, Birmingham, West Midlands, UK; 6 Department of Oncology, University of Oxford, Oxford, Oxfordshire, UK; 7 Surgical Research Laboratory, Institute of Cancer & Genomic Science, University of Birmingham, Birminghaam, United Kingdom; 8 Centre for Cancer Research and Cell Biology, Queen's University Belfast, Belfast, Northern Ireland, UK; 9 Changi General Hospital, Singapore; 10 South Australian Health & Medical Research Institute & School of Medicine, The University of Adelaide, Adelaide, South Australia, Australia

**Keywords:** colorectal cancer, Wnt signalling, stratification, *AXIN2*, molecular biomarker

## Abstract

**Objective:**

Pathological Wnt pathway activation is a conserved hallmark of colorectal cancer. Wnt-activating mutations can be divided into: i) ligand-independent (LI) alterations in intracellular signal transduction proteins (*Adenomatous polyposis coli*, β-catenin), causing constitutive pathway activation and ii) ligand-dependent (LD) mutations affecting the synergistic R-Spondin axis (*RNF43*, *RSPO*-fusions) acting through amplification of endogenous Wnt signal transmembrane transduction. Our aim was to exploit differential Wnt target gene expression to generate a mutation-agnostic biomarker for LD tumours.

**Design:**

We undertook harmonised multi-omic analysis of discovery (n=684) and validation cohorts (n=578) of colorectal tumours collated from publicly available data and the Stratification in Colorectal Cancer Consortium. We used mutation data to establish molecular ground truth and subdivide lesions into LI/LD tumour subsets. We contrasted transcriptional, methylation, morphological and clinical characteristics between groups.

**Results:**

Wnt disrupting mutations were mutually exclusive. Desmoplastic stromal upregulation of *RSPO* may compensate for absence of epithelial mutation in a subset of stromal-rich tumours. Key Wnt negative regulator genes were differentially expressed between LD/LI tumours, with targeted hypermethylation of some genes (*AXIN2*, *NKD1*) occurring even in CIMP-negative LD cancers. *AXIN2* mRNA expression was used as a discriminatory molecular biomarker to distinguish LD/LI tumours (area under the curve >0.93).

**Conclusions:**

Epigenetic suppression of appropriate Wnt negative feedback loops is selectively advantageous in LD tumours and differential *AXIN2* expression in LD/LI lesions can be exploited as a molecular biomarker. Distinguishing between LD/LI tumour types is important; patients with LD tumours retain sensitivity to Wnt ligand inhibition and may be stratified at diagnosis to clinical trials of Porcupine inhibitors.

**Significance of this study**

**What is already known on this subject?**

- Precision medicine requires simple and accurate stratification of patients in advance of treatment decisions.
- The Wnt pathway can be activated either through constitutive activation of downstream signal transduction (ligand-independent (LI) mutation) or from the disruption of the synergistic R-Spondin axis (ligand-dependent (LD) mutation).
- Preclinical models of LD tumours retain therapeutic sensitivity to ligand inhibition, and small molecule Porcupine inhibitors are in early phase clinical trials.

**What are the new findings?**

- Wnt ligand-dependent and ligand-independent tumour subgroups exhibit non-overlapping transcriptional, epigenetic, morphological and clinical characteristics.
- We identify a rare tumour subset, termed *RSPO*-high with Wnt disruption predominantly driven by desmoplastic stromal expression of R-Spondin ligands.
- Differential expression of some Wnt negative regulator genes between tumour subgroups is associated with targeted promoter methylation in LD tumours.
- One of these differentially expressed negative regulators, *AXIN2,* can be used as a discriminatory molecular biomarker of LD tumours with an area under the curve >0.93.

**Significance of this study**

**How might it impact on clinical practice in the foreseeable future?**

- Currently, identification of solid tumour patient subgroups with LD mutations would require multi-omic analysis which is impractical for routine clinical use.
- Use of AXIN2 as a single gene, mutation-agnostic biomarker on diagnostic biopsies could help stratify patients that might benefit from Wnt ligand inhibition.

## Introduction

The Wnt pathway is critical for intestinal development and adult tissue cell fate determination.^1^ Consistent with this key homeostatic role, epithelial Wnt signalling is strictly regulated, with activity predominantly restricted to the bottom half of the intestinal crypt where the adult intestinal stem cell niche and the proliferative transit amplifying cells are located.^2^

The canonical Wnt ligands are predominantly expressed by stem cell niche constituents including telocytes,^3^ *Gli1*+ve fibroblasts^4^ and Paneth cells.^5^ These paracrine morphogens are secreted following palmitoylation by the membrane bound O-acyltransferase, Porcupine^6^ and this post-translational modification is required for activity of Wnt ligands. Wnt activation results from the binding of ligand to cognate *Frizzled (FZD*) and *lipoprotein receptor-related protein* receptors on the cell surface. Recently, the R-Spondin signalling pathway has emerged as a key, co-evolved pathway for Wnt amplification. In the absence of R-Spondin ligand, cell-surface Wnt *FZD* receptors are degraded by the activity of the E3 ubiquitin ligases *Ring finger protein 43 (RNF43*) and *Zinc and Ring Finger 3 (ZNRF3*). Binding of secreted R-Spondin morphogens to transmembrane *leucine-rich repeat-containing G protein coupled receptors 4–6* inhibits RNF43/ZNRF3, disinhibiting Fzd receptors and powerfully amplifying Wnt signalling activity without affecting endogenous canonical Wnt ligand tone.^7^

The key intracellular signal transducer of the canonical Wnt pathway is β-catenin, encoded by *Catenin-Beta1* (*CTNNB1*). In the absence of Wnt ligand, cytosolic β-catenin is targeted for degradation by a multi-protein destruction complex consisting of *Adenomatous polyposis coli (APC), Axin-like protein (Axin 1/2), Glycogen synthase kinase (GSK3β*) and *Casein kinase* (*CK1α*). Ligand-receptor binding blocks the activation of the destruction complex, releasing β-catenin which accumulates in the cytosol, translocates to the nucleus and forms a transcriptional complex, activating a large number of Wnt target genes.

In adult intestinal homeostasis, precise control over the polarised gradient of Wnt signalling activity is vital to permit homeostatic cell function, while avoiding the significant pathological consequences of disrupted Wnt activity. Autoregulatory control of the pathway is exerted by negative feedback loops and these control and fine-tune the physiological Wnt rheostat. These loops are rapidly activated following Wnt pathway stimulation, and act at multiple levels of the signalling cascade including: secreted antagonists (eg, *secreted FZD-related proteins*, ligand palmitoleoylate hydrolases, eg, *Notum palmitoleoyl-protein carboxylesterase (NOTUM*), receptor inhibitors, eg, *Dickkopf-related protein* (*DKK*), *Adenomatosis polyposis coli downregulated 1* (*APCDD1*) and signal transduction inhibitors, eg, *AXIN1/2*, *Naked Cuticle 1 (NKD1*)). Animal models show varying developmental and pathological consequences of Wnt inhibitor knockout, reflecting the context dependency of Wnt signalling function in different cell types.^8^ It is this evolved complexity of the Wnt regulatory network that permits context-dependent amplification or attenuation of Wnt signal activity.

Loss of control of this strictly regulated pathway is associated with pathology in many organs. In the intestine, pathological Wnt activation is a conserved hallmark of intestinal cancer with a spectrum of activating alterations seen in the vast majority of colorectal cancers (CRC).^9^ These include loss-of-function mutations in *APC* and *RNF43* and gain-of-function mutations in *RSPO* (characterised by gene fusions) and *CTNNB1.* 9–11 While *APC* and *CTNNB1* alterations drive downstream activation of the Wnt pathway that is independent of Wnt ligand binding (ligand-independent (LI)), *RSPO* and *RNF43* alterations disrupt the synergistic R-Spondin axis and amplify endogenous Wnt ligand signalling (ligand-dependent (LD)). These mutations are almost always mutually exclusive in CRC, which is consistent with previous work demonstrating that an optimal but not excessive level of Wnt activation is considered favourable for tumourigenesis—the ‘just-right’ theory.^12^

Interestingly, different Wnt pathway mutations are selectively and preferentially acquired in different polyp subtypes. Thus, conventional tubular and tubulovillous adenomas are characterised by *APC* or, less commonly, *CTNNB1* mutations while serrated polyps such as sessile serrated lesions (SSL) and traditional serrated adenomas (TSA) commonly select for *RNF43* mutation or *RSPO* fusions, suggesting that tumours with these mutations follow a distinct evolutionary trajectory.^13^ Proactive molecular stratification of LD or LI Wnt driver mutations is potentially clinically important; tumours driven by LD Wnt driver mutations retain sensitivity to Wnt inhibition through Porcupine inhibitors or anti-RSPO antibodies.^14^ However, there is no simple biomarker available to identify the LD patient cohort which currently requires undertaking targeted panel/exome sequencing, together with RNA sequencing for *RSPO* fusions. Here, we show that differential Wnt negative feedback target gene expression between LD and LI tumours can be exploited to generate a mutation-agnostic, simple, single molecular biomarker to discriminate between tumours, and that this could be used clinically to identify patients with cancers that might be sensitive to preoperative Porcupine inhibitor therapy.

## Methods

Multi-omic profiling performed in each patient cohort is summarised in online supplementary table 1. See online supplementary methods for details on data access, sample selection, nucleic acid extraction, quantitative real-time PCR (qRT-PCR), bioinformatic analyses, in situ hybridisation (ISH) and digital pathology.

10.1136/gutjnl-2019-319126.supp3 Supplementary data

10.1136/gutjnl-2019-319126.supp2 Supplementary data

## Results

### Cohort characteristics

As detailed in table 1.

**Table 1:** Cohort characteristics for the seven cohorts of samples included in this study

Cohort		Origin	Type	Preparation	Sample size	Pathology	Mean age	M:F ratio
*Discovery*	Polyps	Internal (S:CORT)	Biopsy	Fresh/frozen	n=54	Adenoma	67.0	1.00
	A (DC-A)	TCGA^9^	Resection	Fresh/frozen	n=618	Carcinoma (colon, rectum)	66.4	1.15
	B (DC-B)	Genentech^11^	Resection	Fresh/frozen	n=66	Carcinoma (colon)	NA	NA
	Pancancer	TCGA^17^	Resection	Fresh/frozen	n=10 542	Carcinoma (non-colorectal)	59.1	0.92
*Validation*	A (Val-A)	Internal (S:CORT)	Resection	FFPE	n=348	Carcinoma (colon, rectum)	63.5	1.79
	B (Val-B)	Internal (S:CORT)	Biopsy	FFPE	n=230	Carcinoma (rectum)	67	1.71
*Clinical application*		Internal	Resection	FFPE	n=63	Carcinoma (rectum)	70.4	1.63

FFPE, formalin-fixed paraffin embedded; NA, not available; S:CORT, Stratification in Colorectal Cancer.

### Driver Wnt pathway alterations are mutually exclusive in colorectal polyps, colorectal tumours and non-colorectal tumours

To characterise the landscape of Wnt-altering events in CRC and other tumours, we undertook multi-omic analysis of cohorts from the Stratification in Colorectal Cancer (S:CORT) study and publicly available data (methods). The aim was to segregate tumours into two clusters: LI tumours with identifiable mutations in *APC* or *CTNNB1* and LD lesions with *RSPO2/3* fusions or *RNF43* alterations. *APC*, *RNF43* and *CTNNB1* mutations were identified by DNA sequencing. *RSPO2* and *RSPO3* gene fusions were identified by the presence of specific fusion breakpoint sequences in RNA sequencing reads.

**Figure 1:**
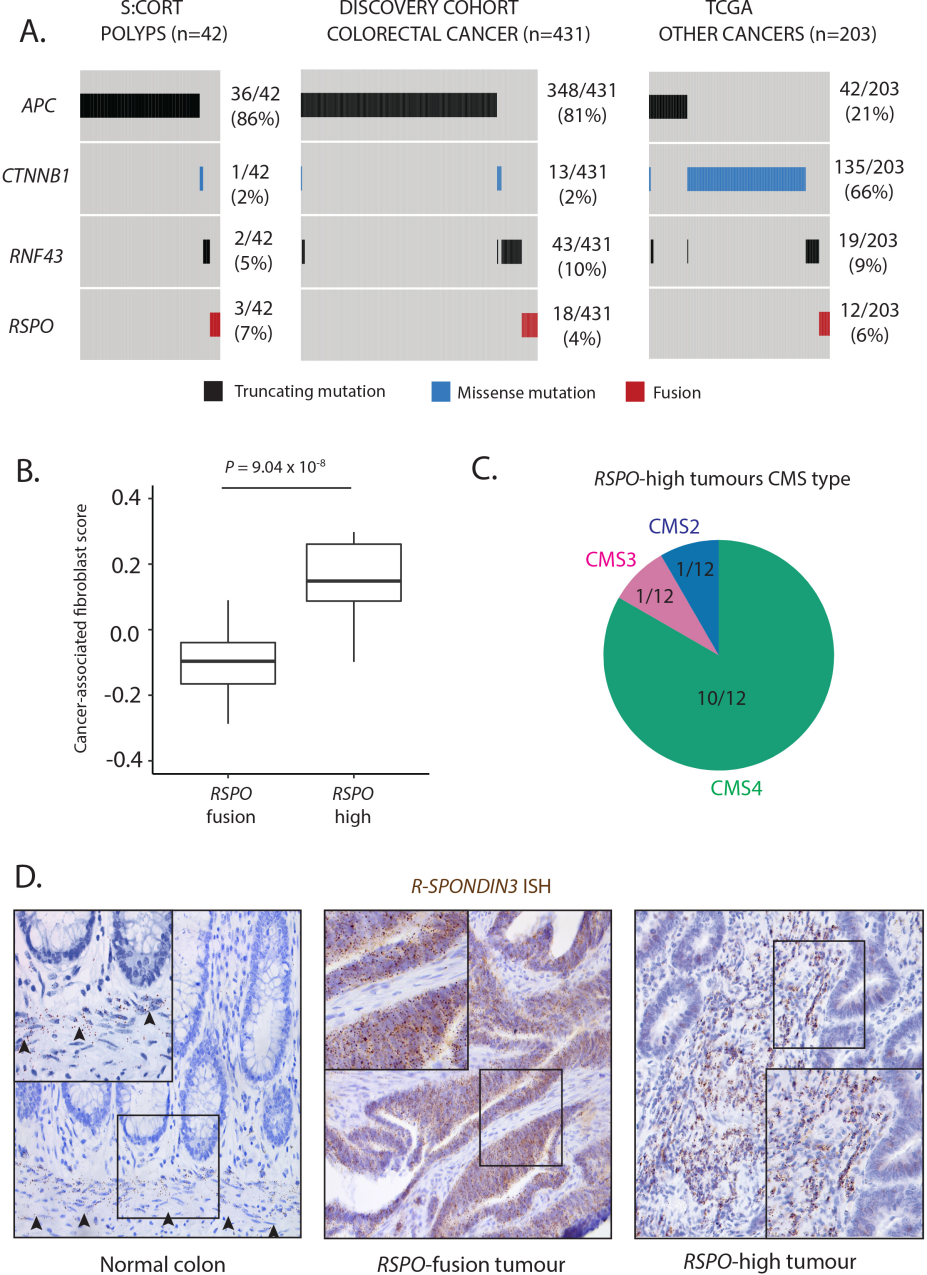
Wnt mutation burden and RSPO-high tumours. (A) Oncoprint of identifiable Wnt disrupting mutation distribution in the Stratification in Colorectal Cancer (S:CORT) polyp cohort, TCGA CRC cohort and TCGA solid cancer cohorts. Only tumours with detectable Wnt alterations are displayed. (B) Comparison of transcriptome-based cancer-associated fibroblast score in RSPO-fusion and RSPO-high tumours. (C) Consensus molecular subtype (CMS) of RSPO-high tumours. (D) RSPO3 in situ hybridisation (brown spots) exclusively from the muscularis mucosae in normal human colon, with aberrant epithelial expression in an RSPO3-fusion tumour and upregulated stromal expression in an RSPO3-high tumour.

Discovery cohorts included a selected precancerous polyp cohort (n=54 from S:CORT), deliberately incorporating polyps from across the histological spectrum (clinicopathological features summarised in online supplementary table 2). Mutational analysis identified activating Wnt alterations in 41/54 polyps (76%) (figure 1A, online supplementary tables 3 and 4). The 13 lesions without detectable mutation were non-dysplastic SSLs—this is consistent with evidence that SSLs acquire Wnt alterations at an advanced stage, as part of progression to dysplasia.^15^ All Wnt alterations identified were mutually exclusive. In line with previous findings,^16^ LD alterations were only identified in TSAs and SSLs, whereas LI alterations were seen across all histological polyp types.

We applied the same stringent mutational analysis to two CRC discovery cohorts (discovery cohort A from TCGA and discovery cohort B from Genentech) incorporating 684 colorectal tumours. Functionally relevant, activating Wnt alterations were identified in 431/684 (63%) tumours (figure 1A, online supplementary tables 3 and 4). In this large tumour cohort, we identified just 14/431 (3%) tumours with more than one detectable Wnt driver alteration.

To assess the spectrum of Wnt drivers in tumours from other primary sites, we undertook mutational analysis across the TCGA pan-cancer cohort of 10 542 non-colorectal tumours^17^ and identified Wnt mutations in 203/10 542 (2%) tumours (figure 1A, online supplementary tables 3 and 4). The most common primary sites for tumours with Wnt disrupting alterations were endometrium, liver and stomach. Consistent with results in our colorectal cohorts, only 4/203 (2%) of non-CRC tumours contained more than one Wnt driver. Together, this mutual exclusivity suggests strong selection pressure against acquiring more than one Wnt driver mutation.

### Stromal overexpression of R-Spondin as a novel driver alteration in colorectal cancer

To internally validate our fusion calling we determined normalised *RSPO2/3* expression in samples with and without *RSPO* fusions. In the polyp cohort, we found that all polyps with outlier *RSPO* expression (defined as z-score ≥2) had detectable *RSPO* fusions (online supplementary figure 1). In contrast, in the discovery cohort, we identified a subset of tumours (termed *RSPO*-high, n=16/684) with outlier expression of *RSPO2/3* despite absence of detectable fusion mutation (online supplementary figure 1). The majority of these samples (10/16) had no other identifiable Wnt alterations. Homeostatically, *RSPO* expression exclusively from muscularis mucosae fibroblasts maintains the intestinal crypt stem cell niche, so we reasoned that tumour microenvironmental *RSPO* expression could compensate for the absence of epithelial Wnt mutations in *RSPO*-high tumours. Consistent with a stromal R-Spondin source, analysis of gene expression profiles in *RSPO*-high versus *RSPO*-fusion tumours showed that the former exhibit significant upregulation of a validated cancer-associated fibroblast gene signature^18^ (figure 1B, p=9.04×10^−8^), and that 10/12 *RSPO-*high tumours with available consensus molecular subtype (CMS) classification were CMS4 (mesenchymal) subtype tumours (figure 1C). In addition, blinded expert histopathological review of digital slides identified trends towards increased tumour budding and reduced glandular formation in *RSPO*-high versus *RSPO*-fusion lesions (online supplementary figure 1B).

10.1136/gutjnl-2019-319126.supp1 Supplementary data

As these discovery sets were derived from publicly available datasets, we had no tissue access to check on-slide *RSPO* cell compartment expression. We therefore collated a cohort of tumours with available tissue sections derived from both a clinical application cohort of rectal tumours (n=63, table 1) and validation cohort A (n=348). Tumours with outlier *RSPO3* expression were profiled using both targeted *RSPO3* RNA sequencing to identify fusion genes and ISH to visualise the origin of the *RSPO3* signal in these samples. We identified six tumours with outlier *RSPO3* expression, four of which had detectable *RSPO3* fusions. The *RSPO*-fusion tumours exhibited a tissue compartmental shift in expression with epithelial localisation of transcripts, whereas *RSPO*-high tumours exhibited marked upregulation of *RSPO3* expression from the desmoplastic stroma (figure 1D, online supplementary figure 2). Finally, ISH on a subset of tumours with non-outlier *RSPO3* expression (n=20) demonstrated qualitative correlation between expression and stromal staining, altogether suggesting that *RSPO3* stromal expression in *RSPO*-high tumours reflects an extreme perturbation of constitutive stromal RSPO3 expression (online supplementary figure 2).

In summary, *RSPO*-high tumours characterise a rare tumour subset, with Wnt disruption predominantly driven by desmoplastic stromal overexpression of R-Spondin ligands. We incorporated this subset of tumours in our LD cluster for further analysis. After including this *RSPO*-high cohort as potential LD tumours, the total number of tumours with detectable LD alterations was 75/684 (11.3%).

### Distinct regulation of endogenous Wnt negative feedback loops in ligand-dependent tumours

**Figure 2:**
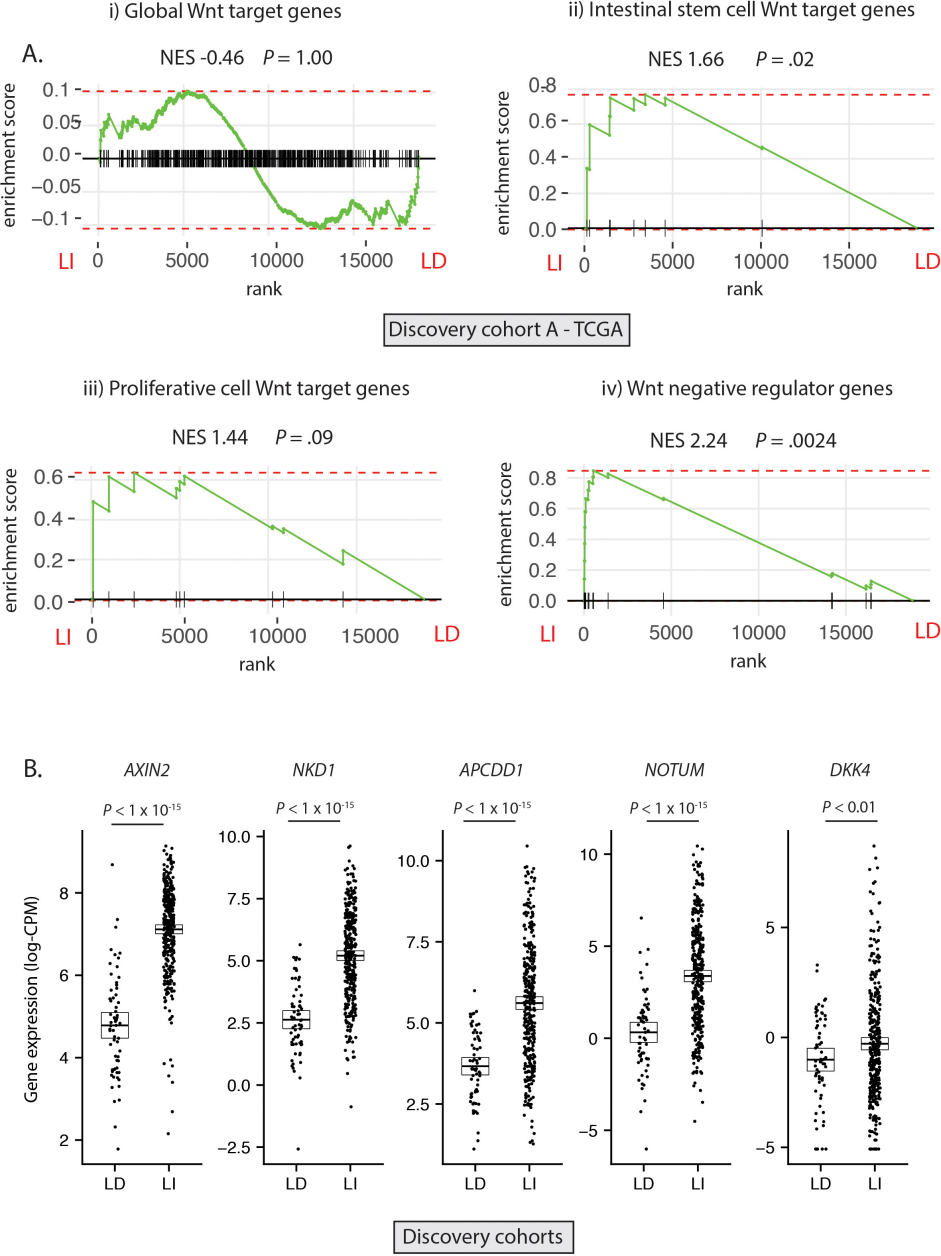
Differential expression of Wnt target genes. (A) Gene set enrichment analysis (GSEA) was performed on discovery cohort A (TCGA) to assess differential expression of i) Global Wnt responsive genes ii) Crypt base columnar stem cell Wnt targets iii) Proliferative cell Wnt targets iv) Wnt negative regulator genes, between ligand-independent (LI) and ligand-dependent (LD) tumours. (B) Gene expression of five leading edge negative regulator (NR) genes between LD and LI tumours in the combined discovery cohorts (log-CPM). NES, Normalised enrichment score.

Having established the molecular ground truth, we segregated tumour samples in the discovery cohort into LD (*RSPO*-fusions, *RSPO*-high and *RNF43* mutations, n=64) and LI (*APC* or *CTNNB1* mutations, n=347) subsets. Samples with concurrent LD and LI alterations (n=9) were excluded to minimise the potential for misclassification (figure 2A). In light of the specific polyp type predilection and mutual exclusivity of different Wnt mutations in colorectal tumourigenesis, we hypothesised that, rather than being interchangeable, LD and LI alterations drive distinct patterns of Wnt pathway activation. To assess this, we performed differential gene expression analysis between LD and LI lesions in our discovery cohorts (online supplementary figure 3A) and undertook gene-set enrichment analysis, incorporating four curated subsets of Wnt target genes (global Wnt responsive, stem cell, proliferative and negative regulator (NR) genes, detailed in online supplementary table 5). While there was no evidence of significant enrichment of global or proliferative Wnt targets in either cohort, there was significant enrichment of NRs (normalised enrichment score (NES) >2.02, p<0.003) and to a lesser extent, stem cell targets (NES >1.53, p<0.04) in LI tumours (figure 2A, online supplementary figure 3B). Analysis of leading-edge genes within the NR signature identified five consensus differentially expressed NRs in the two discovery cohorts, all expressed at a significantly higher level in LI versus LD tumours*—AXIN2, NKD1, APCDD1, NOTUM* and *DKK4* (figure 2B).

### Hypermethylation of endogenous negative regulators in ligand-dependent tumours

**Figure 3:**
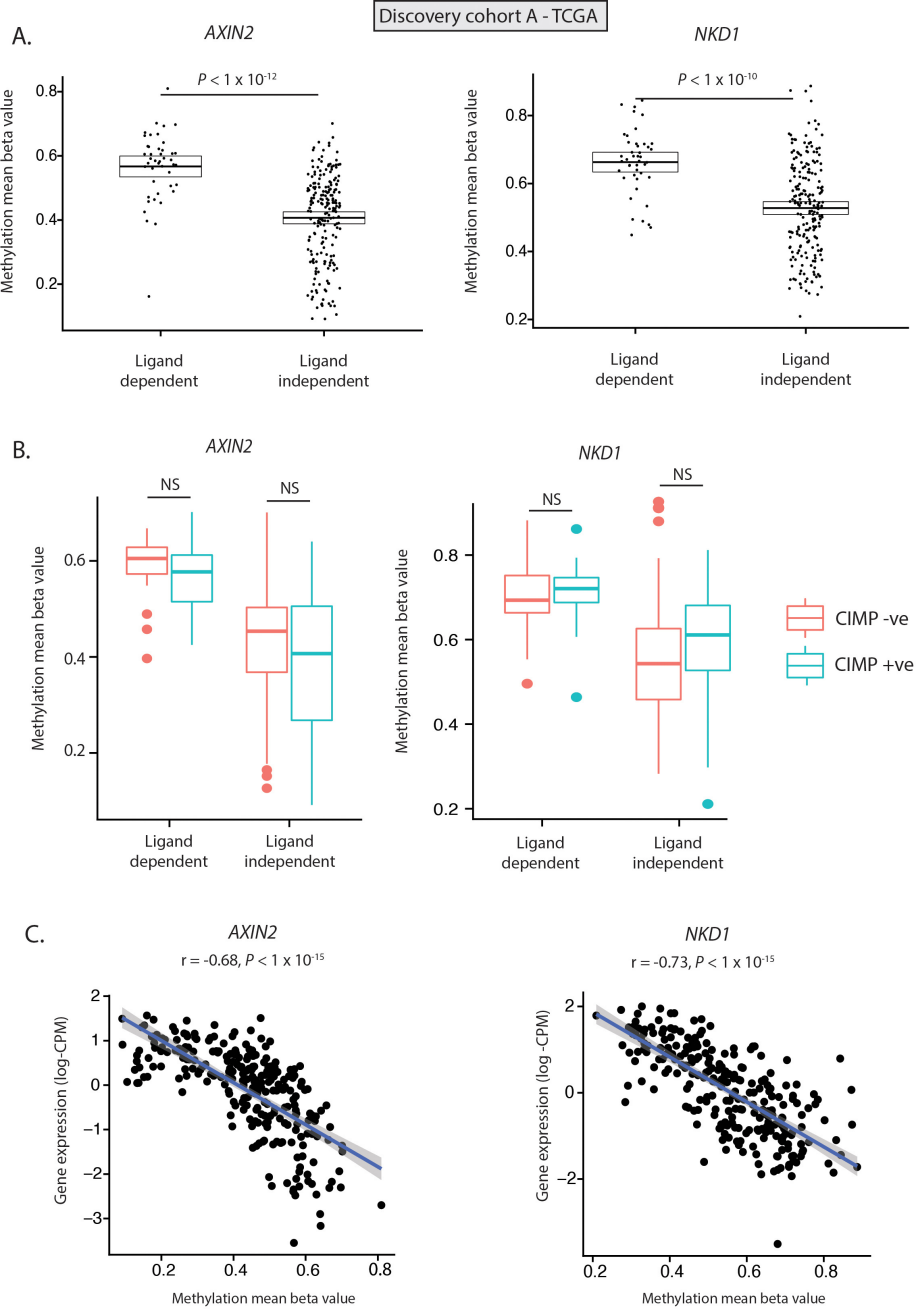
Methylation of key negative regulator genes. (A) Methylation (mean beta value) of AXIN2 and NKD1 showing differential methylation of Wnt NR genes between LD and LI tumours. (B) Methylation (mean beta value) of AXIN2 and NKD1 shows no significant difference between CpG island methylator phenotype (CIMP) positive (blue), and negative (red) LD and LI tumours. (C) Significant anticorrelation between AXIN2 and NKD1 normalised gene expression (log-counts per million/CPM) and gene methylation (mean beta value). All data from discovery cohort A (TCGA).

Previous studies have identified evidence of hypermethylation of Wnt NRs, such as *AXIN2* and *DKK1*, in serrated lesions.^19^ As a result, we hypothesised that differential expression of Wnt NRs could be driven by differential methylation. To investigate this, we undertook methylation analysis of tumours in discovery cohort A (n=440)—the only discovery cohort with available methylation data. We identified 26 differentially methylated probes (DMPs) annotated to four of the five differentially expressed Wnt NR genes (*AXIN2, NKD1, APCDD1, NOTUM*) (online supplementary table 6); 25/26 of these probes were significantly hypermethylated (online supplementary table 6), with significantly elevated mean beta values for each of the four NR genes in LD versus LI tumours (figure 3A, online supplementary figure 4). To understand whether this signal was derived entirely from aberrant methylation in CpG island methylator phenotype (CIMP) tumours, we determined mean beta value for each NR in both CIMP+ve and CIMP−ve LD and LI tumour subsets. Interestingly, while hypermethylation of *NOTUM* and *APCDD1* was associated with CIMP positivity (online supplementary figure 4), there was no significant difference in methylation of *AXIN2* and *NKD1* between CIMP+ve and CIMP−ve tumours, indicating CIMP-independent selective methylation of these genes (figure 3B). Consistent with a functional impact of hypermethylation, *AXIN2* and *NKD1* exhibit significant and near-linear anticorrelation between methylation and normalised gene expression (figure 3C, r=−0.68 and r=−0.72 respectively, online supplementary table 7). In contrast, *NOTUM* and *APCDD1* exhibit a bimodal distribution of methylation beta value, implicating a role for alternative regulatory mechanisms (online supplementary figure 4). Together these data suggest that hypermethylation of endogenous Wnt NRs, especially *AXIN2* and *NKD1,* may act in a synergistic fashion with LD Wnt disrupting mutations.

### Application of *AXIN2* as a clinical biomarker for ligand-dependent tumours

**Figure 4:**
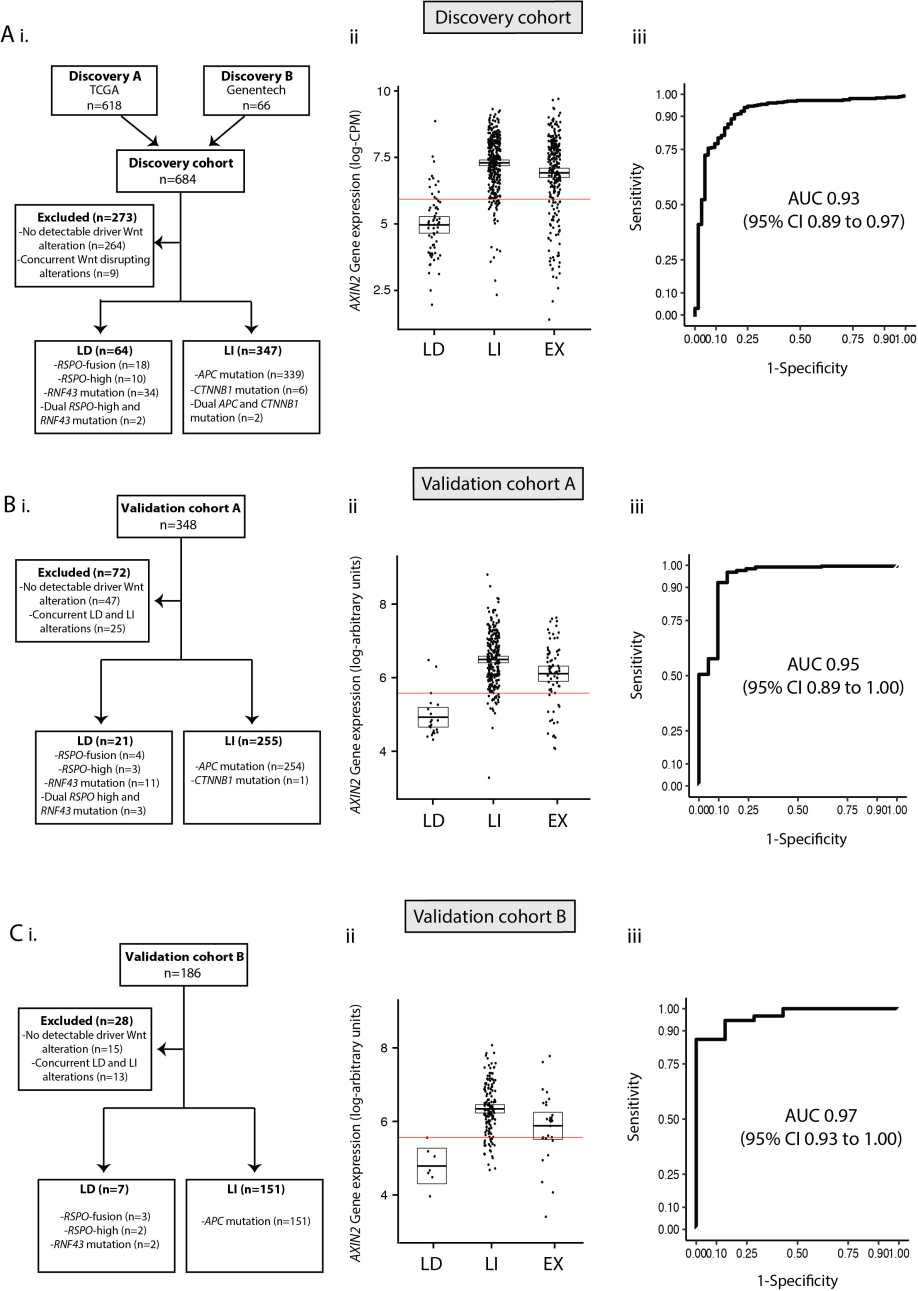
AXIN2 as a clinically discriminatory biomarker. (A) Combined discovery cohorts (TCGA and Genentech). (B) Validation cohort A (S:CORT). (C) Validation cohort B (S:CORT). i) Flow diagram showing exclusion criteria. ii) Differential AXIN2 mRNA expression between LD and LI tumours. iii) Receiver operating characteristic (ROC) curve to assess diagnostic performance of AXIN2 expression. AXIN2 thresholds shown by red lines in combined discovery cohorts, validation cohort A and validation B were 5.75 log-CPM, 5.58 log-arbitrary units and 5.57 log-arbitrary units respectively.

Having demonstrated consistent, differential expression of Wnt NRs, we hypothesised that we could exploit these discriminatory differences to identify and validate a simple, mutation-agnostic biomarker to identify patients with LD tumours that could potentially benefit from Porcupine inhibition. First, we applied receiver operating characteristic (ROC) curve analysis to the LD (n=64) and LI (n=347) subsets within the discovery cohort to determine the diagnostic utility of each consensus NR (online supplementary table 8). This analysis showed that *AXIN2* demonstrated superior diagnostic performance with area under the curve (AUC) of 0.93 (95% CI 0.89 to 0.97, figure 4A). Using an *AXIN2* expression threshold to maximise the sum of sensitivity and specificity, this translated to sensitivity and specificity of 94.5% and 78.5%, respectively (threshold=5.75 log-counts per million/CPM).

Next, to validate the accuracy of *AXIN2* as a feasible biomarker for identification of LD tumours, we turned to two validation cohorts (Val-A/Val-B) derived from the S:CORT study (table 1). Using molecular ground truth data, each cohort was stratified into three subsets: LD, LI or unclassified (termed excluded (EX), figure 4B, C). In both cohorts, differential *AXIN2* expression was discriminatory between LD and LI tumours while EX tumours exhibited variable *AXIN2* expression (figure 4B, C). ROC curve analysis in these validation sets confirmed the LD/LI discriminatory capacity of *AXIN2* expression with AUC of 0.95 (95% CI 0.89 to 1.00) in Val-A (figure 4B) and 0.97 (95% CI 0.93 to 1.00) in Val-B (figure 4C). Using an *AXIN2* expression threshold to maximise the sum of sensitivity and specificity, this translated to sensitivity and specificity of 92.2% and 90.4% in Val-A (threshold=5.58 log-arbitrary units) and 86.1% and 100% in Val-B (threshold=5.57 log-arbitrary units).

To further evaluate *AXIN2* as a tumour biomarker, we wanted to assess both qRT-PCR and immunohistochemistry (IHC) as alternative approaches for scoring *AXIN2* expression in a realistic clinical setting. We extracted RNA from one formalin-fixed paraffin embedded (FFPE) section per lesion from our clinical application cohort (n=63), and immunohistochemically stained a subsequent section for AXIN2 protein. RNA was assayed using both 3’RNA-sequencing and qRT-PCR using the Fluidigm platform. In our hands, only 31/63 of these FFPE samples passed stringent quality control for 3’RNA-sequencing (aligned reads >75%) in contrast to 62/63 samples for qRT-PCR. There was robust correlation between *AXIN2* scored by qRT-PCR and 3’RNA-sequencing (n=31, r=0.75, p=1.73×10^−6^, online supplementary figure 5). Next, we scored AXIN2 IHC staining on available sections (n=53) using two complementary approaches: automated scoring using the HALO digital pathology platform and manual scoring by expert histopathologist in a random subset of tumours (n=27). Across the cohort, we identified weak correlation between qRT-PCR metrics of *AXIN2* mRNA expression and manual scores of AXIN2 IHC (r=0.35, p=0.077, online supplementary figure 5), while there was no significant correlation with automated scores of AXIN2 IHC (p>0.29). However, the four tumours with known LD status (*RSPO-*fusion n=3, *RSPO*-high n=1) had comparably low manually scored AXIN2 protein expression (online supplementary figure 5). In summary, qRT-PCR for *AXIN2* is a feasible approach to assay *AXIN2* expression in a situation of limited tissue availability, while IHC staining is insufficiently sensitive to consistently reflect differences in *AXIN2* transcriptome expression.

### Ligand-dependent disease positioning in colorectal cancer

In light of evidence that LD tumours derive from distinct precursor lesions and have different Wnt pathway dynamics, we hypothesised that there would be conserved clinicopathological differences between LD and LI tumours, and detectable differences between subsets with distinct LD alterations. To investigate this, we pooled tumours with available clinicopathological annotations (discovery cohort A and validation cohort A, n=635). We then contrasted demographics (age, sex, tumour location), molecular pathology (*BRAF/KRAS* mutation status, microsatellite instability, CMS subtype), computationally scored histopathology (mucin, stroma, tumour and glandular area per slide) and prognostic (overall survival, disease-free survival (DFS)) metrics for each subtype.

**Figure 5:**
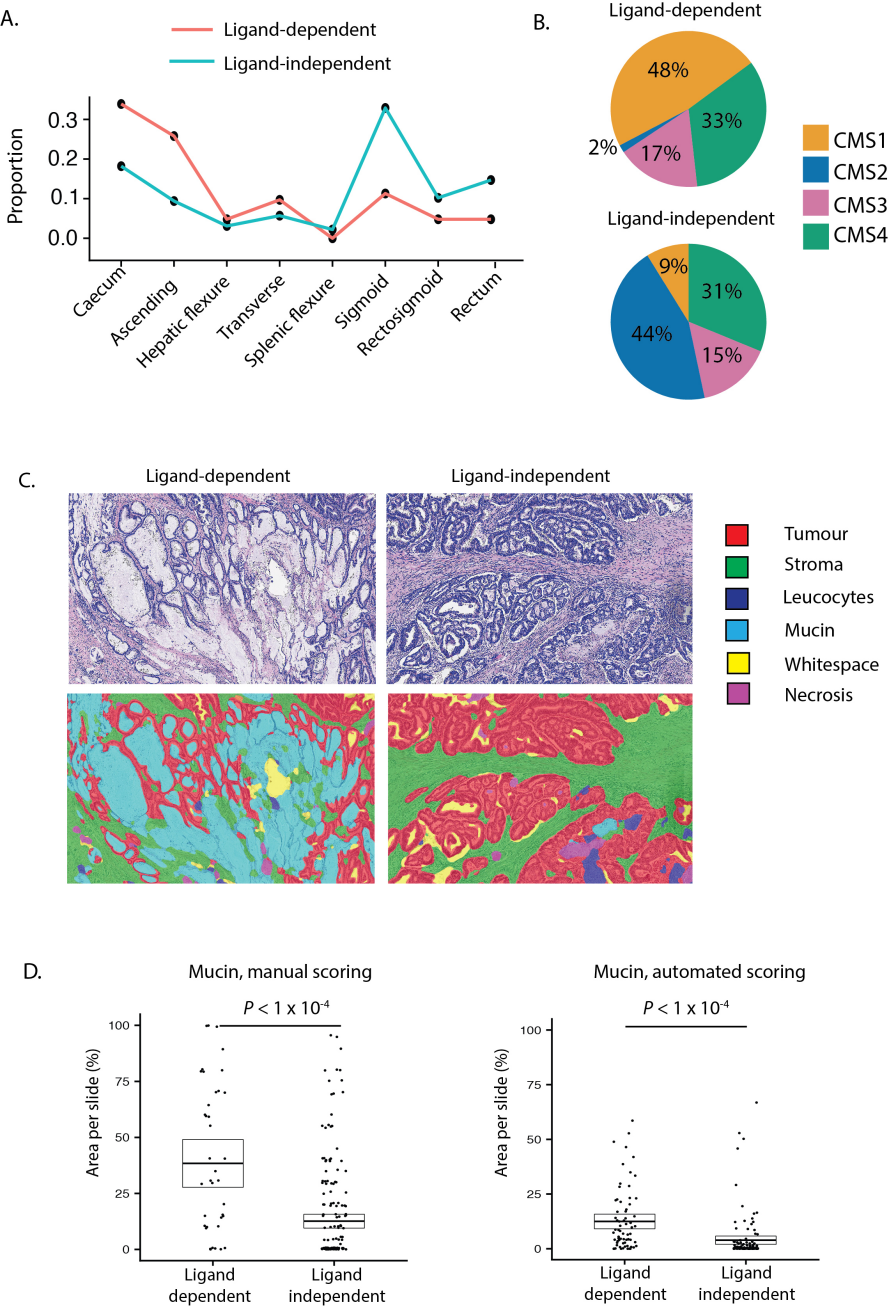
Clinical disease positioning of ligand-dependent and independent tumours. (A) Colonic distribution proportion of ligand-dependent (LD) (red line) and ligand-independent (LI) (blue line) tumours. (B) Consensus molecular subtype (CMS) of LD and LI tumours. (C) Representative images from digital pathology supervised image segmentation of invasive cancer into tissue compartments. (D) Manual and computational scoring of mucin area in LD and LI tumours segregated by molecular ground truth data.

In comparison to LD tumours, LI tumours were significantly enriched for left-sided tumours (sigmoid and rectum), *KRAS* mutations and CMS2 (canonical) classification, consistent with conventional lesion molecular pathway (figure 5A, B, online supplementary table 9). Analysis of prognostic data identified a non-significant trend towards worse DFS in LI tumours (HR=1.42, p=0.0594). LD tumours were also substratified into *RSPO*-fusion, *RSPO*-high and *RNF43*-mutant subsets. To minimise misclassification of tumours, samples with both outlier *RSPO2/3* expression and *RNF43* mutations (n=6) were excluded leaving a total of 68 tumours. *RNF43*-mutant tumours were significantly enriched for right colonic distribution, *BRAF* mutations, microsatellite instability and CMS1 (MSI immune) classification, consistent with predominantly sessile serrated lesion aetiology (online supplementary table 10). Conversely, 50% of *RSPO*-fusion tumours were located distal to the sigmoid, with a lower frequency of CMS1, suggesting enrichment of tumours progressing from traditional serrated adenoma precursors (online supplementary figure 5C).

Unexpectedly, using automated segmentation of available scanned tumour sections (n=194) we found that computationally scored mucin area was significantly increased in LD tumours (p=2.02×10^−5^, figure 5C). To validate this finding, we extracted published^20^ manually scored mucin area data for a subset of tumours in DC-A (n=246) and confirmed this metric is also markedly increased in LD tumours (p=2.40×10^−5^, figure 5D). To assess the degree to which mucin area can distinguish between LD and LI tumours, we performed ROC curve analysis for each mucin metric. This demonstrated AUC of 0.78 (95% CI 0.70 to 0.85) for computationally scored mucin and AUC of 0.75 (95% CI 0.66 to 0.83) for manually scored mucin.

## Discussion

The ability to segregate colorectal tumour patients into clinically meaningful subsets is important to identify patients suitable for targeted therapies and allows comparison and correlation of molecular, morphological and clinical phenotypes. Wnt pathway disruption through selected (epi)genetic mutation is an obligate requirement in almost all CRCs and is common in many other types of solid tumours. However, accurate stratification of patients and successful therapeutic manipulation of pathological Wnt signalling has proven challenging. The majority of colorectal tumours are Wnt ligand-independent, activating the pathway constitutively, through mutation of the intracellular signal transduction machinery. However, a significant subset select for epithelial mutations affecting the synergistic *R-Spondin* axis, rendering them sensitive to therapeutic manipulation of canonical ligand expression. Here, we have also identified a small, but potentially interesting cohort of tumours that lack epithelial Wnt driver mutations altogether—instead profoundly upregulating *R-Spondin* signalling from the tumour stroma. This suggests that stromal compartment expression of Wnt ligands can compensate for absence of selected epithelial mutation in these predominantly aggressive, mesenchymal-subtype (CMS4), ‘*RSPO*-high’ tumours. To identify patients with convergent epithelial or stromal LD Wnt disruption, we demonstrate variable expression of Wnt responsive genes in CRC subsets, and use this to highlight differential *AXIN2* expression as a simple, discriminatory and mutation-agnostic molecular biomarker.

**Figure 6:**
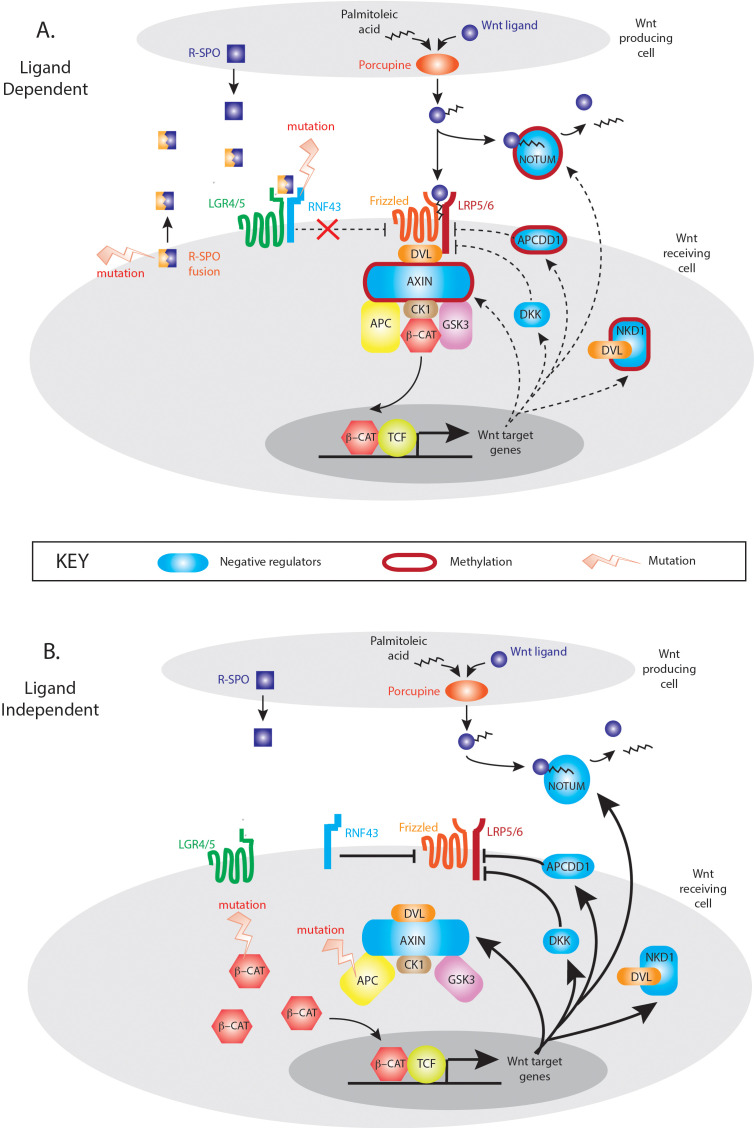
Model of Wnt negative regulation in ligand-dependent (LD) and ligand-independent (LI) tumours. In LD tumours, mutations in the synergistic RSPO axis upregulate Wnt signalling through an otherwise intact canonical Wnt signalling pathway. In this situation, appropriate upregulation of Wnt NRs (blue shapes) could restrain pathological Wnt pathway activity which generates a selective pressure for targeted methylation and epigenetic suppression of key Wnt NR activity (red borders). In LI tumours, downstream mutation in APC and CTNNB1 causes constitutive activation of Wnt intracellular signalling pathways rendering Wnt negative feedback functionally redundant and disconnecting the physiological negative regulator equilibrium.

The mutual exclusivity of LD and LI mutations suggests that activation of either the *R-Spondin* or downstream canonical axis of Wnt signalling alone is sufficient for tumourigenesis. Multiple Wnt driver mutations result in excessive Wnt disruption and are selected against the ‘just-right’ hypothesis. However, despite this mutual exclusivity, Wnt disrupting mutations are not necessarily equivalent with respect to downstream target gene activation, with differences in Wnt responsive gene enrichment between LD and LI tumours. The Wnt responsive gene set that showed maximal divergence was ‘NRs’, with five significantly differentially expressed genes between LD and LI tumours. These important autoregulatory genes are rapidly responsive to Wnt stimulation and act in parallel, at multiple pathway levels to control the intensity and/or duration of the signal. Expression divergence in these tumour subsets may be partly explained by epigenetic regulation, with hypermethylation of NRs in some LD tumours. Gene methylation correlates with low transcript expression, and Aza-C demethylation treatment restores *AXIN2* expression in a dose-dependent fashion in CRC cell lines.^21^ Interestingly, epigenetic suppression of two key Wnt NRs (*AXIN2*, *NKD1*) in LD tumours can occur independently of the CpG island methylator phenotype, where promoter methylation is globally dysregulated.^22^ This suggests that targeted, rather than stochastic methylation of Wnt regulatory genes may occur in these lesions. This is biologically plausible. Wnt activation in LD tumours is the consequence of increased flux through an otherwise intact intracellular signalling pathway. Physiologically relevant and functioning negative feedback loops would constrain pathological LD Wnt signalling, so epigenetic downregulation of involved NR genes would be advantageous in lesions progressing down a LD molecular pathway. In contrast, the constitutive, intracellular activation of Wnt signalling through *APC* or *CTNNB1* mutation in LI tumours, renders upstream Wnt NRs functionally redundant and uncouples appropriate feedback loop equilibrium (figure 6). The mechanistic explanation for these observations needs further detailed functional research, but the differential methylation and expression of key Wnt target genes across the spectrum of LD and LI tumours should be considered when using genes like *AXIN2* as a surrogate measure of global Wnt activation.

LD tumours retain sensitivity to Wnt ligand inhibition with Porcupine inhibitor^14^ or anti-RSPO3 antibody therapy^23^ resulting in regression and differentiation of human *RSPO*-fusion tumours in patient-derived xenograft models. Our analysis across tumour types has detected LD Wnt alterations in colorectal (10%), stomach (2%), endometrial (1%) and pancreas (1%) tumours while recent work has identified a subset of hepatocellular carcinomas (1%) with *RSPO2* gene fusions.^24^ Detection of these alterations at diagnosis might allow these patients to benefit from chemotherapeutic Wnt ligand suppression. Using current techniques, LD tumour discrimination requires genetic and transcriptomic analysis of all tumours to identify Wnt disrupting mutations and *RSPO*-fusion transcripts. This is impractical for routine diagnostic assessment. We have exploited the differential expression of Wnt responsive genes across the tumour spectrum to identify a molecular biomarker that discriminates LD/LI lesions at the target gene level. *AXIN2* was the most discriminant single biomarker, with AUC for mRNA expression >0.93 in three independent cohorts. Quantitative assessment of *AXIN2* mRNA expression was unaffected by the type of sequencing technique (PolyA RNA seq, 3’RNA seq or qRT-PCR), and could be assessed with high discriminatory yield from diagnostic endoscopic biopsy samples in a validation cohort. This supports potential clinical application of the biomarker to stratify patients from endoscopic samples at the point of diagnosis, and in advance of treatment decisions. There was limited correlation between differential RNA expression and the heterogenous IHC staining of AXIN2 protein in our clinical application samples. This may be related to tissue processing variability or antibody insensitivity but known post-translational regulation and altered degradation of AXIN2 protein could also contribute.^25^ This precludes the use of IHC staining assessment as a standard diagnostic tool to identify LD patient subgroups.

Across the tumour cohorts in this study, there was a large number of lesions without identifiable Wnt disrupting alterations. This may result partly from missed mutations in retrospective data sets, for example, *RNF43* hotspot mutations can be difficult to capture as they are susceptible to microsatellite loci slippage. However, this does not exclude the possibility of rare, novel epithelial (epi)genetic Wnt drivers, or a cell-extrinsic, microenvironmental Wnt source—as seen in *RSPO*-high lesions. The range of *AXIN2* expression seen in the EX lesions here, suggests that *AXIN2* could be used to direct the search towards new LD or LI drivers.

Wnt signalling has remained difficult to target in solid tumours, because of developmental and homeostatic importance, pathway complexity and the frequency of non-actionable, signal transduction mutations. Although uncommon, LD tumours represent an opportunity for therapeutic Wnt inhibition and effective small molecule inhibitors are in phase I trials for a range of solid malignancies.^26^ Precision targeted therapies rely on detection of suitable and distinct patient cohorts. Here, we have demonstrated molecular and phenotypic distinction between Wnt disrupted CRC tumour populations and exploited differential Wnt target expression to generate a simple, transcriptome-based biomarker to identify patients with LD tumours for entry into stratified medicine clinical trials.
