# Supplementary material for: Exploiting differential Wnt target gene expression to generate a molecular biomarker for colorectal cancer stratification
Source: Gut. 2019 Sep 28;69(6):1092–103. doi: 10.1136/gutjnl-2019-319126 (PMC7212029; doi:10.1136/gutjnl-2019-319126)
Supplement: Supplementary data [file gutjnl-2019-319126supp002.pdf]

SUPPLEMENTAL METHODS

Data access

For discovery cohort A and pan-cancer cohort, read counts per gene, RNA sequencing BAM files and tissue slide images were downloaded from the Genomic Data Commons Portal, mutation calls were downloaded from COSMIC, CIMP status was extracted from published data[1] while clinicopathological data (including MSI status) and methylation data was downloaded from the Broad GDAC FIrehose. For discovery cohort B, DNA sequencing and RNA sequencing FASTQ files from datasets EGAD00001000215 and EGAD00001000216 were downloaded from the European Genome-phenome Archive. For all cohorts (except clinical application cohort), samples were excluded if paired mutation and gene expression data were not available.

Sample selection

For all cohorts (except clinical application cohort), samples were excluded if paired mutation and gene expression data were not available. For internal cohorts, all samples were obtained following ethical approval and individual informed consent (Ethics No. 15/EE/0241 for S:CORT cohorts and Ethics No. 09/H0606/5+5 for clinical application cohort). For the pre-cancer cohort polyps were selected across the histopathological spectrum. All internal samples were subject to expert histopathological review.

Patient and public involvement

This work was supported by a patient and public involvement and engagement sub-group led by Mark Lawler. The sub-group met regularly and organised a number of patient group engagement meetings to discuss the S:CORT programme of work.

Nucleic acid extraction

For internal cohorts of frozen samples, RNA was isolated with RNeasy Mini Kit (Qiagen) and DNA was isolated with DNA QIAamp Mini Kit (Qiagen). For internal cohorts of FFPE samples, 2-5x10 micron sections were cut; RNA was isolated with High Pure FFPET RNA Isolation Kit (Roche) and DNA was isolated with High Pure FFPET DNA Isolation Kit (Roche).

DNA sequencing analysis

Sequencing reads were mapped to GRCh37 plus decoy (GRCh37d5) using BWA-MEM[2]. Substitution and base insertion/deletions were identified using CaVEMan[3] and Pindel[4] respectively. For samples without a paired normal, mutations were called against a representative unmatched normal and normal variants flagged for removal. Variants located in regions covered by the bait design were then annotated using Variant Effect Predictor[5]. Truncating mutations were defined as frameshift, nonsense or essential splice variants. Our stringent mutational analysis solely included truncating *APC*, truncating *RNF43* mutations and missense *CTNNB1* mutations affecting codons 31–35, 37, 40, 41, 45, 383 and 387. For polyps cohort and validation cohorts A-B, MSI status was determined by analysis of 123 microsatellite regions included in the targeted panel and tumours were classified as MSI if ≥3 regions contained an insertion or deletion.

DNA methylation analysis

Probe-level beta value data derived from Illumina Infinium HumanMethylation450 (HM450) platform was analysed using the ChAMP methylation pipeline for R[6]. Probes were filtered using default parameters and normalized using peak-based correction. Differentially methylated probes were selected on arbitrary absolute delta beta value >0.1 and adjusted p-value <0.05 (Benjamini-Hochberg correction).

RNA sequencing analysis

Sequencing reads were mapped using STAR[7] in two-pass mode. For human RNA-seq, reads were aligned to GRCh37, except for discovery cohort A which was mapped to GRCh38. Expression counts per gene were determined by HTSeq[8] or STAR and strand-specific data was extracted where appropriate. Expression data was then processed using the edgeR package for R using TMM normalisation[9] with data from multiple cohorts batch-corrected using the the limma package for R[10]. Differentially expressed genes were identified using a generalised linear model-likelihood ratio test and filtered based on an arbitrary absolute $\log_2$ fold change >1 and adjusted p-value <0.05 (Benjamini-Hochberg correction).

RSPO fusion analysis

In cohorts with traditional RNA-seq available, we screened for *RSPO* fusions by searching for 8 previously-validated breakpoint motifs[11–13] (Supplementary Table 11). This approach was validated using the unfiltered output of STAR-Fusion[14] reads for all positive calls with available data and a random subset of negative calls, as well as published fusion calls[11,15]. In cohorts without traditional RNA-seq, targeted RNA sequencing was performed on samples with outlier *RSPO3* expression (defined as ≥2 SDs from the mean) using a custom QIAseq Targeted RNAscan panel (Qiagen), with fusions identified by motif search.

mRNA expression microarray analysis

mRNA microarray was performed using the Affymetrix Almac Xcel Arrayl. Raw intensity data was log-transformed, processed and normalized (RMA) using limma[10] and affy[16] for R. Due to the altered *RSPO* transcript structure in samples with *RSPO* fusions, one *RSPO3* probe (ADXEC.2609.C1_s_at) was selected on the basis of Spearman's correlation between RNA-seq and Xcel microarray data in the polyps cohort. For all other genes, mean probe expression per gene was used.

Quantitative real-time PCR (qRT-PCR)

To determine *AXIN2* and *RSPO3* expression in our clinical application cohort, we utilized the high-throughput Fluidigm Biomark HD Real-Time PCR platform (Fluidigm). cDNA was pre-amplified using the Fluidigm Preamp Master Mix and run on a 192.24 IFC chip. Each reaction was performed in quadruplicate and the expression of each target was normalized to endogenous controls (*EEF1A1, ACTB, GAPDH*). Primer sequences are detailed in supplementary table 12.

Gene expression signatures

Cancer-associated fibroblast signature expression was scored by single-sample gene set enrichment analysis (ssGSEA) performed using the GSVA package for R[17]. GSEA for Wnt target genes was performed using the fgsea package for R[18]. Consensus molecular subtypes[1] were determined using the CMSclassifier package for R.

Immunohistochemistry

Automated staining was carried out with the Leica BOND-MAX autostainer (Leica, Microsystem) using the following conditions: antigen retrieval at 100ºC for 20 min with Epitope Retrieval Solution 2 (AR9640, Leica Biosystems), primary antibody incubation with the AXIN2 antibody (Abcam, ab32197) at 1:2000 dilution or IgG control for 30mins and detection using the BOND™ Polymer Refine Detection System (DS9800, Leica Biosystems) as per manufacturer's instructions.

***In situ* hybridization**

In situ hybridization was performed using the RNAscope 2.0 HD Detection Kit (Brown) for FFPE (Advanced Cell Diagnostics, probe Hs-RSPO3-O3 (491461)).

Digital pathology

All digital slides underwent histopathology review with tumour budding defined per ITBCC[19] and mucinous histology defined according to WHO criteria. Invasive cancer tissue was annotated using the HALO™ image analysis platform version 2.2.1870.6 (Indica Labs). Supervised image segmentation was performed using Random Forest classification. Results were re-reviewed using digital mark-up overlays in conjunction with the original slide to assure accuracy. For immunohistochemical scoring, average optical density in each of four segmented tissue areas and two cellular compartments (nuclear/cytoplasmic) was determined to output 8 metrics. Orthogonal validation was performed by manual expert histopathology scoring on a subset of samples to generate a combined epithelial expression score as % positive cells x intensity of expression (0-3). All histopathological assessments were blinded to the underlying molecular ground truth.

Statistical analysis

Receiver operating characteristic (ROC) curves were generated using the pROC package for R[20]. 95% confidence intervals were determined by 10,000 replicates of stratified bootstrap analysis. Significant difference was taken at the $p<0.05$ level using Welch's t tests unless otherwise stated.

SUPPLEMENTAL REFERENCES

1 Guinney J, Dienstmann R, Wang X, *et al.* The consensus molecular subtypes of colorectal cancer. *Nat Med* 2015;**21**:1350–6. doi:10.1038/nm.3967

2 Li H, Durbin R. Fast and accurate short read alignment with Burrows-Wheeler transform. *Bioinformatics* 2009;**25**:1754–60. doi:10.1093/bioinformatics/btp324

3 Jones D, Raine KM, Davies H, *et al.* cgpCaVEManWrapper: Simple Execution of CaVEMan in Order to Detect Somatic Single Nucleotide Variants in NGS Data. *Curr Protoc Bioinformatics* 2016;**56**:15.10.1-15.10.18. doi:10.1002/cpbi.20

4 Ye K, Schulz MH, Long Q, *et al.* Pindel: a pattern growth approach to detect break points of large deletions and medium sized insertions from paired-end short reads. *Bioinformatics* 2009;**25**:2865–71. doi:10.1093/bioinformatics/btp394

5 McLaren W, Gil L, Hunt SE, *et al.* The ensembl variant effect predictor. *Genome Biol* 2016;**17**:122. doi:10.1186/s13059-016-0974-4

6 Morris TJ, Butcher LM, Feber A, *et al.* Champ: 450k chip analysis methylation pipeline. *Bioinformatics* 2014;**30**:428–30. doi:10.1093/bioinformatics/btt684

7 Dobin A, Davis CA, Schlesinger F, *et al.* STAR: ultrafast universal RNA-seq aligner. *Bioinformatics* 2013;**29**:15–21. doi:10.1093/bioinformatics/bts635

8 Anders S, Pyl PT, Huber W. HTSeq — a Python framework to work with high-throughput sequencing data. *Bioinformatics* 2015;**31**:166–9. doi:10.1093/bioinformatics/btu638

9 Robinson MD, McCarthy DJ, Smyth GK. edgeR: a Bioconductor package for differential expression analysis of digital gene expression data. *Bioinformatics* 2010;**26**:139–40. doi:10.1093/bioinformatics/btp616

10 Ritchie ME, Phipson B, Wu D, *et al.* limma powers differential expression analyses for RNA-sequencing and microarray studies. *Nucleic Acids Res* 2015;**43**:e47. doi:10.1093/nar/gkv007

11 Seshagiri S, Stawiski EW, Durinck S, *et al.* Recurrent R-spondin fusions in colon cancer. *Nature* 2012;**488**:660–4. doi:10.1038/nature11282

12 Sekine S, Yamashita S, Tanabe T, *et al.* Frequent PTPRK-RSPO3 fusions and RNF43 mutations in colorectal traditional serrated adenoma. *J Pathol* 2016;**239**:133–8. doi:10.1002/path.4709

13 Sekine S, Ogawa R, Hashimoto T, *et al.* Comprehensive characterization of RSPO fusions in colorectal traditional serrated adenomas. *Histopathology* 2017;**71**:601–9. doi:10.1111/his.13265

14 Haas B, Dobin A, Stransky N, *et al.* STAR-Fusion: Fast and Accurate Fusion Transcript Detection from RNA-Seq. *BioRxiv* Published Online First: 24 March 2017. doi:10.1101/120295

15 Klijn C, Durinck S, Stawiski EW, *et al.* A comprehensive transcriptional portrait of human cancer cell lines. *Nat Biotechnol* 2015;**33**:306–12. doi:10.1038/nbt.3080

16 Gautier L, Cope L, Bolstad BM, *et al.* affy--analysis of Affymetrix GeneChip data at the probe level. *Bioinformatics* 2004;**20**:307–15. doi:10.1093/bioinformatics/btg405

17 Hänzelmann S, Castelo R, Guinney J. GSVA: gene set variation analysis for microarray and RNA-

seq data. *BMC Bioinformatics* 2013;**14**:7. doi:10.1186/1471-2105-14-7

18 Sergushichev A. An algorithm for fast preranked gene set enrichment analysis using cumulative statistic calculation. *BioRxiv* Published Online First: 20 June 2016. doi:10.1101/060012

19 Lugli A, Kirsch R, Ajioka Y, *et al.* Recommendations for reporting tumor budding in colorectal cancer based on the International Tumor Budding Consensus Conference (ITBCC) 2016. *Mod Pathol* 2017;**30**:1299–311. doi:10.1038/modpathol.2017.46

20 Robin X, Turck N, Hainard A, *et al.* pROC: an open-source package for R and S+ to analyze and compare ROC curves. *BMC Bioinformatics* 2011;**12**:77. doi:10.1186/1471-2105-12-77
