# Supplementary material for: Exploiting differential Wnt target gene expression to generate a molecular biomarker for colorectal cancer stratification
Source: Gut. 2019 Sep 28;69(6):1092–103. doi: 10.1136/gutjnl-2019-319126 (PMC7212029; doi:10.1136/gutjnl-2019-319126)
Supplement: Supplementary data [file gutjnl-2019-319126supp001.pdf]

SUPPLEMENTAL FIGURES

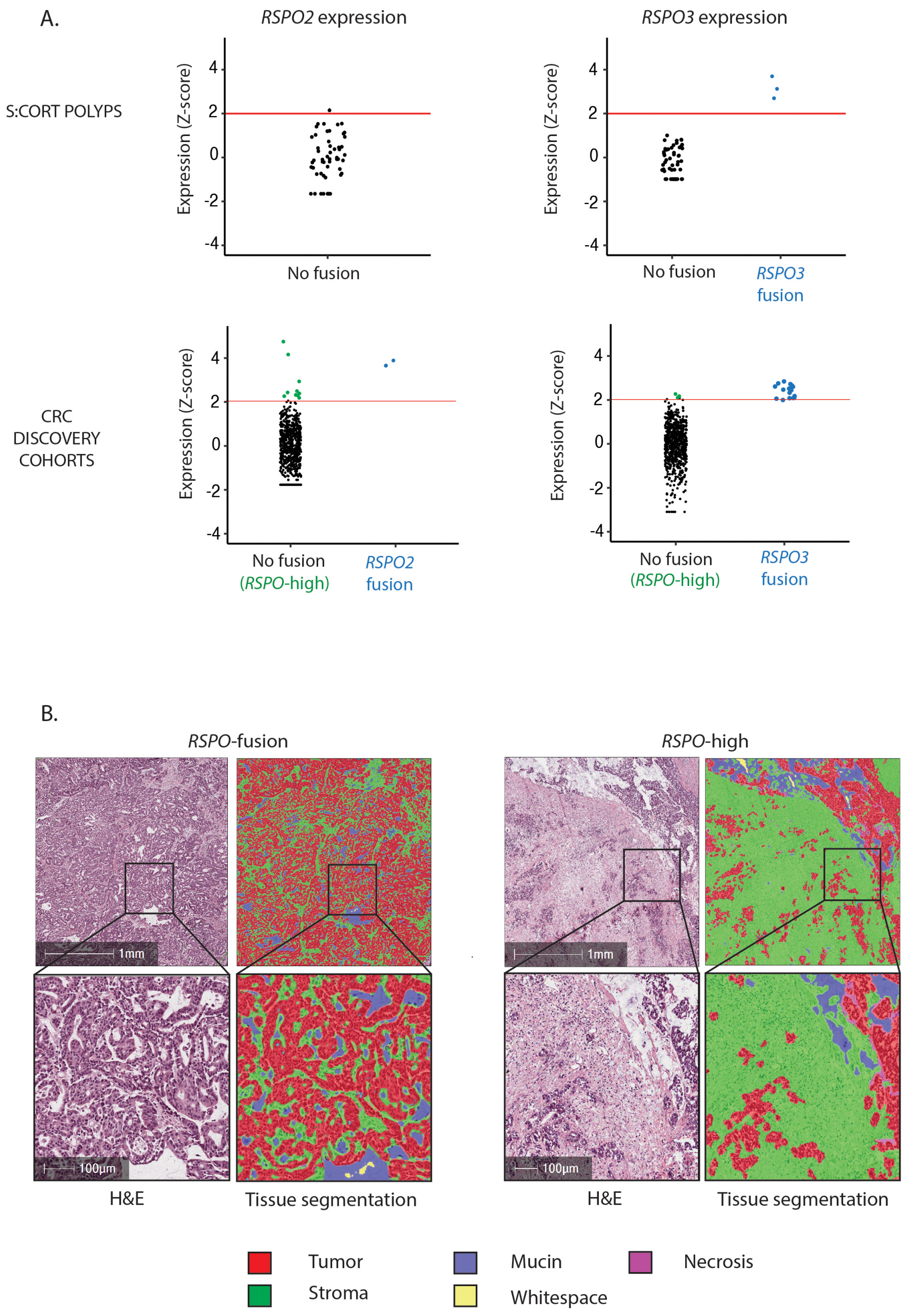

Supplementary Figure 1

A. Expression of *RSPO2/3* and correlation with identified fusion mutations (blue dots) in polyp cohort (n=54) and combined CRC discovery cohorts (n=684). Tumours with outlier *RSPO* expression (Z-score>2) but no evidence of fusion mutation, classified as *RSPO*-high (green dots). B. Representative images contrasting the morphological characteristics and tissue segmentation of *RSPO*-fusion and *RSPO*-high tumours, demonstrating reduced glandular differentiation and increased tumour budding in *RSPO*-high tumours.

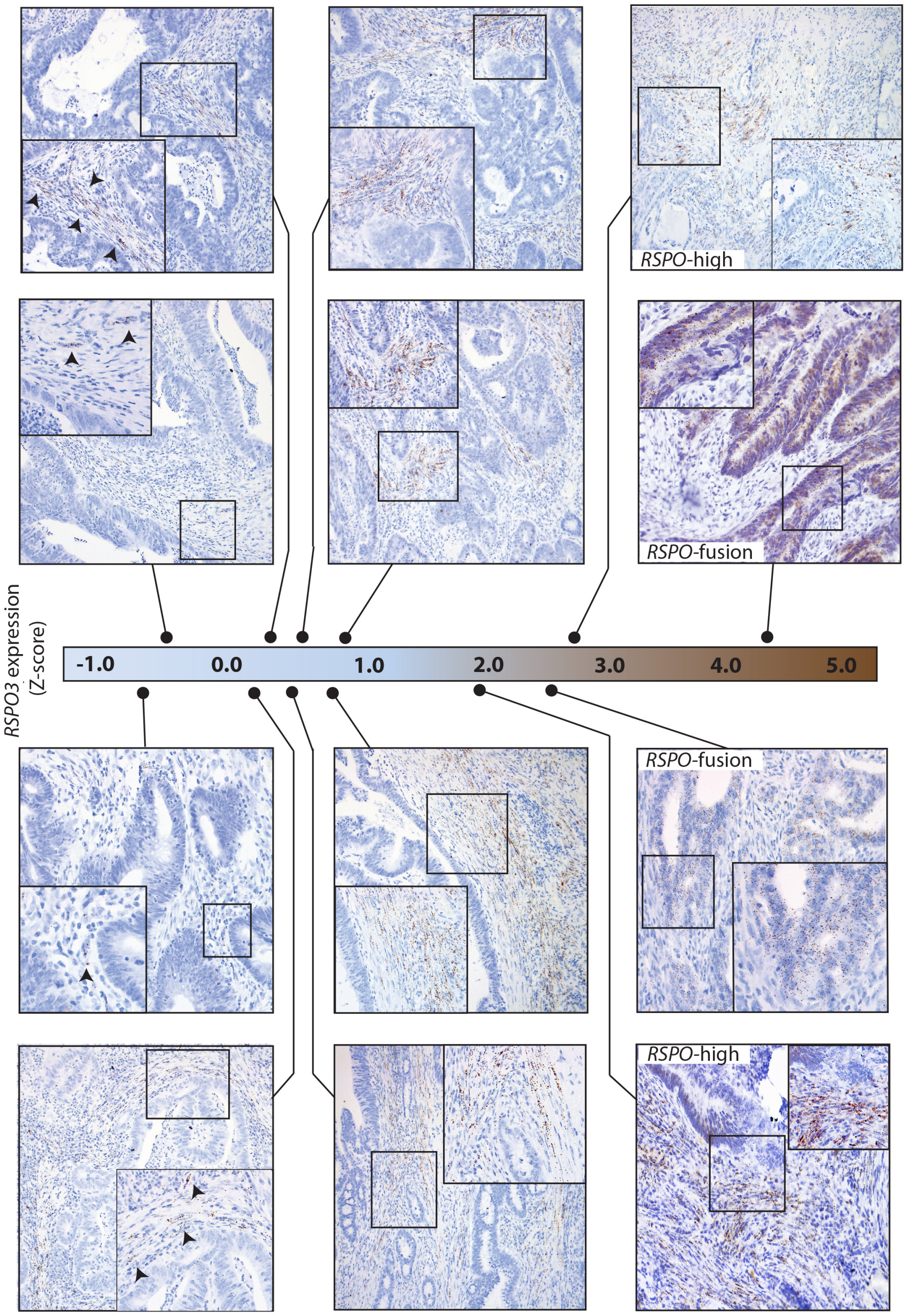

Supplementary Figure 2

RNA *in situ* hybridization (ISH) performed on colorectal tumours across dynamic range of *RSPO3* mRNA expression (indicated as Z-score scale). Tumours with outlier *RSPO3* expression are subdivided into those with (*RSPO*-fusion) or without (*RSPO*-high) detectable *RSPO* fusions. For wild-type tumours there is moderate correlation between mRNA expression and staining (brown spots) in the stromal compartment, with *RSPO*-high tumours reflecting an extreme perturbation of constitutive stromal *RSPO3* expression.

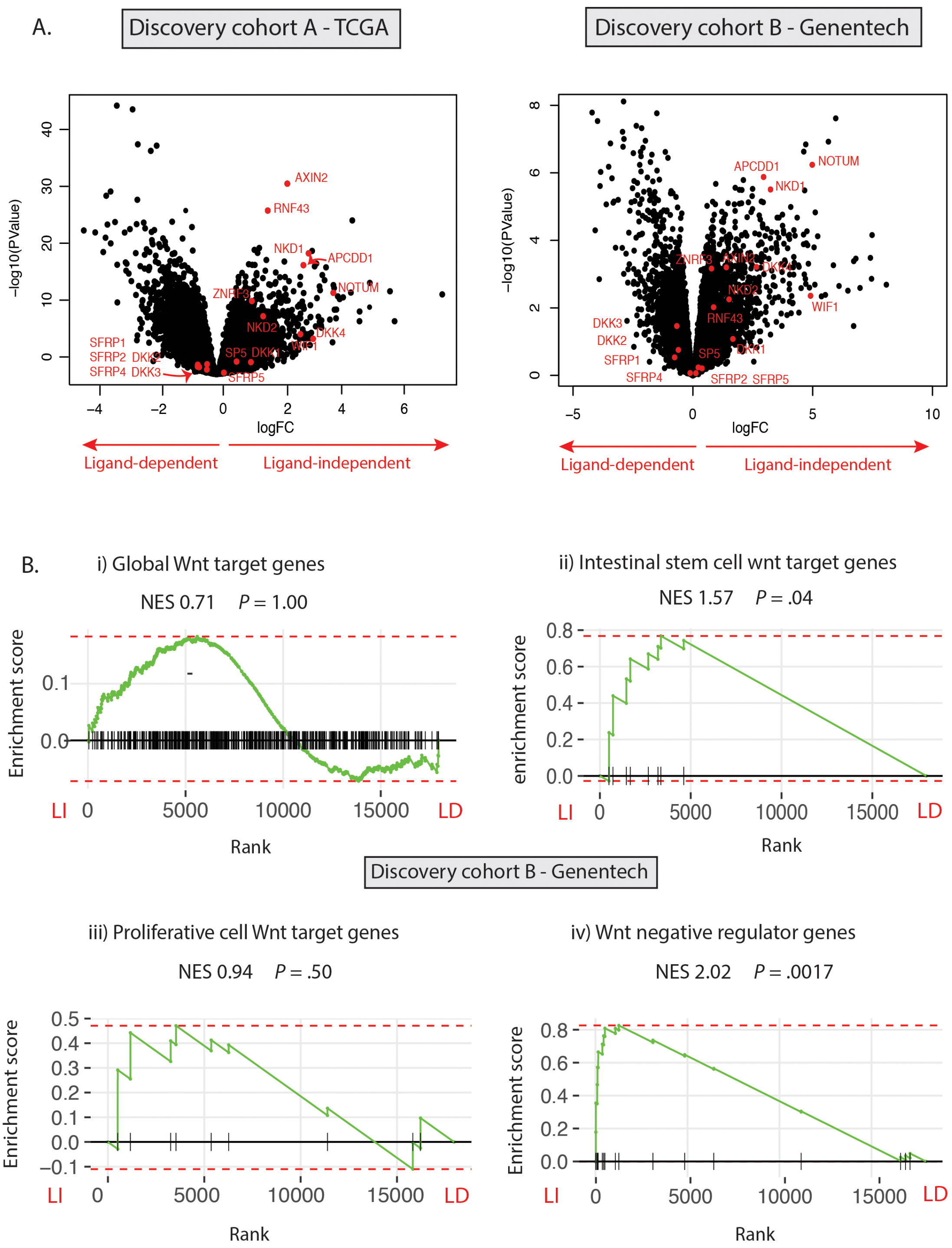

Supplementary Figure 3

A. Volcano plots showing differential expression of genes between LD and LI tumours in discovery cohort A (TCGA) and discovery cohort B (Genentech) with NRs labelled in red. B. GSEA was performed on discovery cohort B (Genentech) to assess differential expression of i) Global Wnt responsive genes ii) Crypt-base columnar stem cell Wnt targets iii) Proliferative cell Wnt targets iv) Wnt NR genes, between LI and LD tumours.

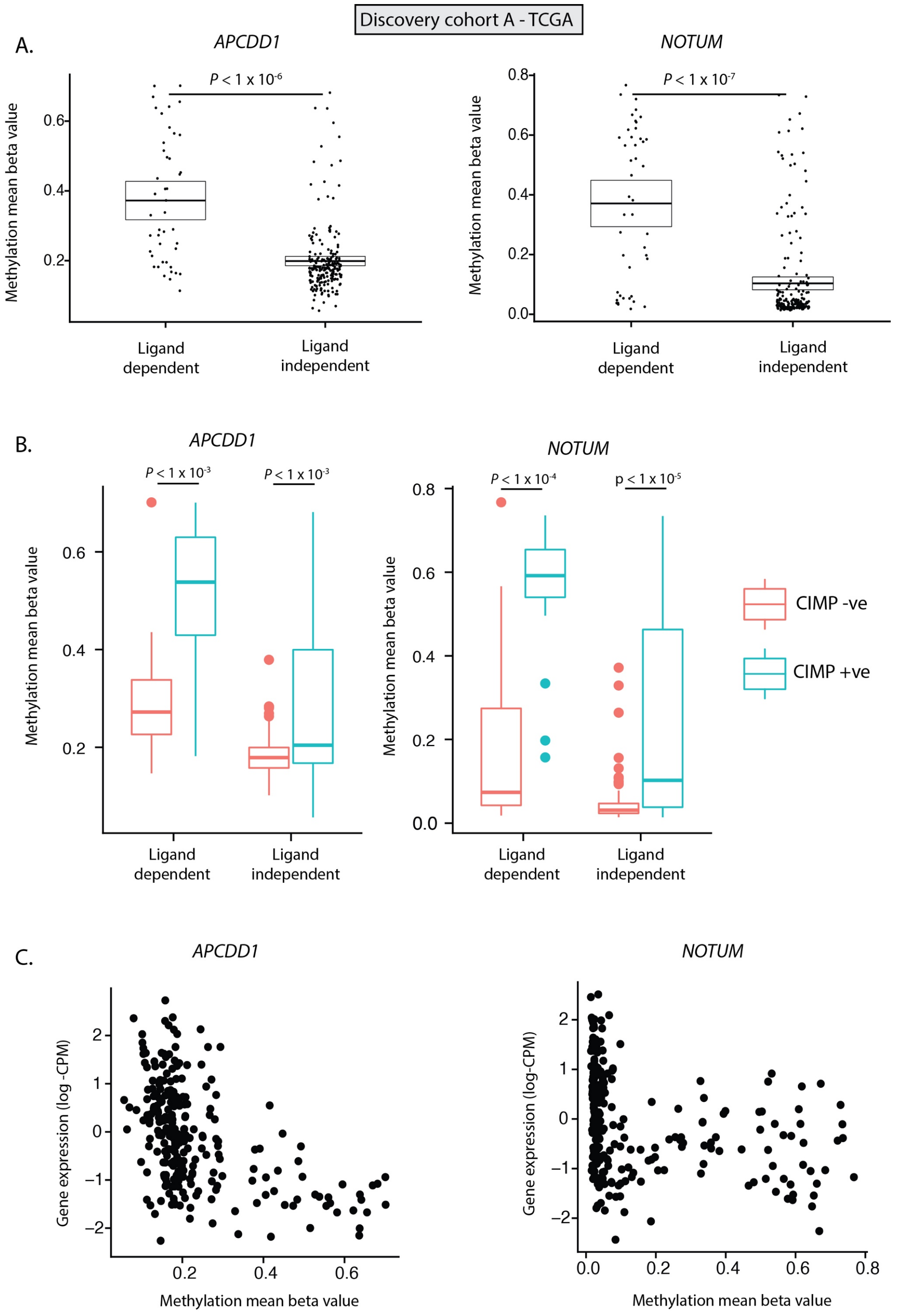

Supplementary Figure 4

A. Methylation (mean beta value) of *APCDD1* and *NOTUM* showing differential methylation of Wnt negative regulator genes between LD and LI tumours. B. Methylation (mean beta value) of *APCDD1* and *NOTUM* shows significant difference between CIMP positive (blue), and negative (red) LD/LI tumours, indicating that these genes undergo methylation as part of the dysregulation of genome-wide methylation associated with CIMP. C. Significant anticorrelation between *APCDD1* and *NOTUM* normalized gene expression (log-CPM) and gene methylation (mean beta value). All data from discovery cohort A (TCGA).

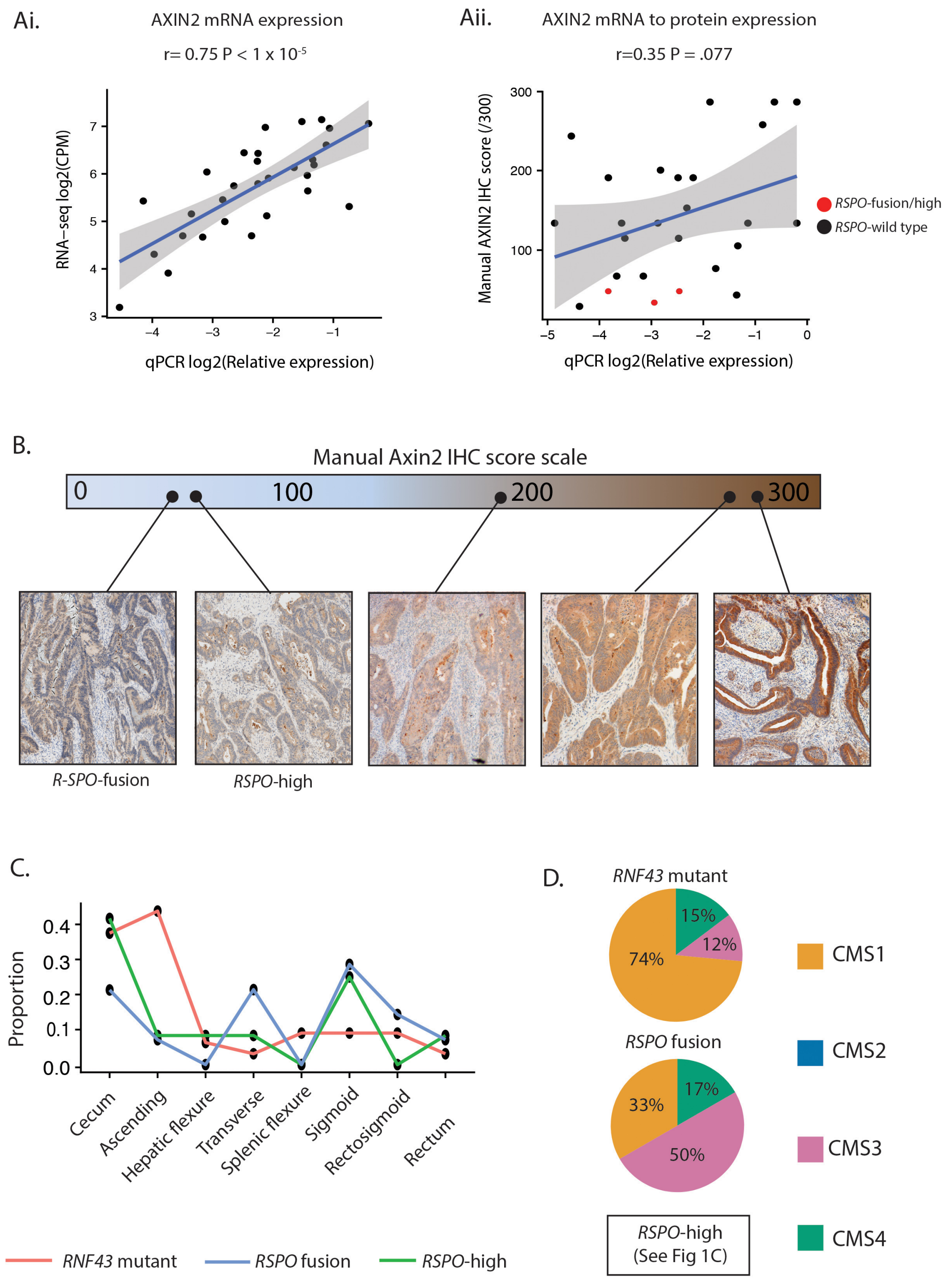

Supplementary Figure 5

A. i) Correlation between *AXIN2* mRNA metrics assessed by 3'RNA Seq and qRT-PCR in clinical application cohort. ii) Correlation between *AXIN2* mRNA assessed by qRT-PCR and manual pathologist scoring of *AXIN2* immunohistochemistry. Red dots indicate known *RSPO*-fusion/high tumours (n=3). One sample with *RSPO*-fusion failed *AXIN2* qRT-PCR and so was excluded from this analysis. B. Comparably low AXIN2 IHC expression in known *RSPO*-fusion and *RSPO*-high tumours from clinical application cohort with representative images of intermediate and high staining across the manual IHC score spectrum. C. Colonic distribution proportion of LD tumours subdivided into *RNF43* mutant (red line), *RSPO*-fusion (blue line) and *RSPO*-high (green line) subsets. D. Consensus molecular subtype of LD tumours subdivided into *RNF43* mutant and *RSPO*-fusion subtypes (see Fig 1C for *RSPO*-high).
